# Epiphysiodesis for the treatment of tall stature and leg length discrepancy

**DOI:** 10.1007/s10354-021-00828-8

**Published:** 2021-03-18

**Authors:** Madeleine Willegger, Markus Schreiner, Alexander Kolb, Reinhard Windhager, Catharina Chiari

**Affiliations:** 1 Department of Orthopedics and Trauma Surgery—Division for Orthopedics, Comprehensive Center for Pediatrics, Medical University of Vienna, Währinger Gürtel 18–20, 1090 Vienna, Austria; 2 Vienna Bone and Growth Center, Vienna, Austria

**Keywords:** Predicted excessive final height, Percutaneus epiphysiodesis, Limb length discrepancy, Skeletal age, Growth prediction, Großwuchs, Perkutane Epiphysiodese, Beinlängendifferenz, Knochenalter, Wachstumsprognose

## Abstract

Painful orthopedic conditions associated with extreme tall stature and leg length discrepancy (LLD) include back pain and adopting bad posture. After failure of conservative treatment options, blocking of the growth plates (epiphysiodesis) around the knee emerged as gold standard in patients with tall stature and LLD in the growing skeleton. Surgical planning includes growth prediction and evaluation of bone age. Since growth prediction is associated with a certain potential error, adequate planning and timing of epiphysiodesis are the key for success of the treatment. LLD corrections up to 5 cm can be achieved, and predicted extreme tall stature can be limited. Percutaneous epiphysiodesis techniques are minimally invasive, safe and efficient methods with low complication rates. In general, a multidisciplinary approach should be pursued when treating children and adolescents with tall stature.

## Abstract (de)

Zu den schmerzhaften orthopädischen Beschwerden, welche mit extremen Hochwuchs und Beinlängendifferenzen (BLD) einhergehen, gehören Rückenschmerzen und schlechte Körperhaltung. Nach Versagen der konservativen Behandlungsmöglichkeiten hat sich die chirurgische Blockade der Wachstumsfugen (Epiphysiodese) um das Kniegelenk als Goldstandard bei Patienten mit Hochwuchs und BLD im wachsenden Skelett etabliert. Die präoperative Planung umfasst die Erstellung einer Wachstumsprognose und die Bewertung des Knochenalters. Da die Wachstumsprognose einer potenziellen Abweichung unterliegt, sind eine angemessene Planung und der adäquate Zeitpunkt der Epiphysiodese (ED) der Schlüssel zum Erfolg. BLD bis zu 5 cm können durch eine ED während des Wachstums korrigiert werden. Ein prognostizierter extremer Hochwuchs kann ebenfalls verringert werden. Perkutane Epiphysiodesetechniken sind minimal-invasive, sichere und effiziente Methoden mit geringen Komplikationsraten. Im Allgemeinen sollte bei der Behandlung von Kindern und Jugendlichen mit Hochwuchs ein multidisziplinärer Ansatz verfolgt werden.

## Introduction

Human growth is a complicated biological activity that is strongly controlled by genetic, hormonal, nutritional and environmental influences. In order to define “normal growth” or “normal height”, one has to take a closer look on the general population. Over the last 200 years an increase in human stature could be observed due to enhancement of the dietary, sanitary, economical and health status of the population [1]. Thus, tall stature is gaining acceptance in our society and could even be considered a benefit. Taller men and women are associated with having more favorable personality traits, greater self-esteem, higher intelligence, and ambitions [2, 3]. Tall stature is not a disease and in general does not need to be treated. However, extreme tallness has been associated with painful orthopedic complaints, including back pain, and poor posture to look shorter. In addition, psychological and psychosocial difficulties, including social withdrawal, and feeling different from peers, play a detrimental role in above average tall children and adolescents, while adults typically face more practical challenges in obtaining adequate clothes, bedding, and shoes [4, 5]. Since the beginning of the 1960s, pediatric endocrinologists have sought to control excessive body size by administering high doses of sex steroids to promote maturation and arrest skeletal growth. While such procedures limited final height to some extent, they were accompanied by severe side effects, including impaired fertility [6, 7].

Leg length discrepancy (LLD) is not only a cosmetic burden but also a functional concern. An increased energy expenditure during walking due to the excessive vertical rise and fall of the pelvis is needed. Long-standing significant discrepancies often lead to back pain and compensatory scoliosis with decreased spinal mobility. As a surgical treatment option, epiphyseodesis of the growth plates about the knee has been shown to be a reliable and safe technique for reducing final height in tall stature and to correct LLD. The most important limiting factor represents the adequate timing of epiphysiodesis. Growth prediction of final height underlies a certain error, which needs to be taken into account for surgical planning and interpretation of surgical outcome.

The present review describes the basic principles of epiphysiodesis, outlines the currently used methods for growth prediction, and summarizes surgical outcomes of epiphysiodesis for tall stature and leg length discrepancy.

## Causes for tall stature

Compared to children with short stature, children with tall stature are rarely referred to a pediatric endocrinologist for evaluation of possible pathologic triggers. Although most tall infants are in good health, there are some syndromes with severe complications which should be ruled out. Therefore, a detailed history should be taken that includes information about the childbirth, the physical examination, and the assessment of the growth chart. The diagnostic workup should be carried out in a multidisciplinary setup together with a pediatric endocrinologist who will evaluate puberty [8]. Among syndromic tall stature, Marfan syndrome but also other rare connective tissue disorders are often characterized by excessive adult height. An increased birth weight and length may lead to the diagnosis of a Beckwith–Wiedemann syndrome, while development deficiencies may be related to the following syndromes: Klinefelter, triple X, fragile X, homocystinuria, Sotos and Weaver. A history of lens luxation can reveal Marfan syndrome or homocystinuria, while cardiovascular defects are characteristic of Marfan syndrome, neonatal hypotonia is seen in Sotos syndrome, and abdominal wall and macroglossia defects can be present in Beckwith–Wiedemann syndrome [9]. Additional skeletal symptoms like scoliosis and joint laxity should lead to cardiac and ophthalmologic evaluation [8, 10].

## Causes for leg length discrepancy

Leg length discrepancy (LLD) is common among the general population. During screening of military recruits differences in leg lengths were observed in 77%, and about 36% had discrepancies greater than 0.5 cm [11]. LLD less than 1 cm is most likely clinically insignificant and can be a variance of the normal. However, LLD greater than 1 cm may lead to stress on the shorter leg and back. Children with LLD may present with an abnormal gait, pelvic obliquity, back pain, differences in knee height, limb size or shoe size. In order to rule out, or detect a possible cause for LLD (Table 1), a history of trauma, infection, neurologic disease, abnormal skin pigmentation, or cutaneous vascular abnormalities may lead to a diagnosis. A thorough orthopedic physical exam is followed by visual gait analysis. Finally, a standing anterior/posterior (a/p) bilateral lower extremity radiograph is taken in order to perform measurements of the actual length of the limb, the femur, and the tibia. The X‑ray should also be checked for additional mechanical axis deviations [12].

**Table 1:** Causes for limb length discrepancy [13]

Congenital	Secondary	Syndromes	Neurologic	Tumor
Developmental dysplasia of the hip (DDH)/dislocated hip	Trauma/fracture (long bone or growth plate fracture)	Klippel–Trenaunay syndrome	Cerebral palsy (hemiplegic)	Benign lesion
Hemimelia (fibula, tibia)	Fracture malunion	Proteus syndrome	Polio	Malignant lesions
Congenital femur deficiency (CFD)	Osteomyelitis/septic arthritis			
Congenital tibial dysplasia (CTD) –anterolateral bowing of the tibia	Perthes disease/avascular necrosis	Parks–Weber syndrome		
Posteromedial/anterior bowing of the tibia	Chronic inflammatory arthritis (rheumatoid, psoriatic, or lupus arthrosis, etc.)	Beckwith–Widemann syndrome		
Clubfoot	Ischemic damage to the growth plate (iatrogenic, vascular impairment, arteriovenous malformations)			
Calcaneal-valgus foot	Mechanical damage to the growth plate (Blount disease)			
	Radiation exposure			
	Scoliosis			

## Height prediction/growth prediction

**Fig. 1:**
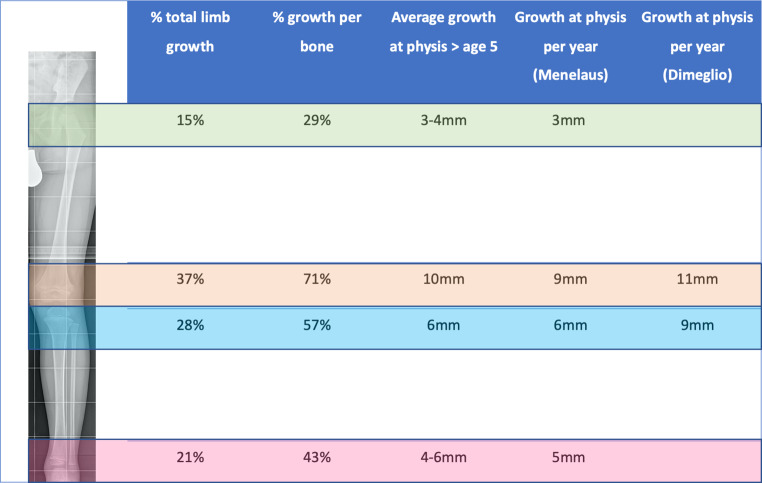
Data outlining the growth of the lower extremity. Overview of current knowledge on lower limb growth adapted from Lovell and Winter’s *Pediatric Orthopaedics*: Lippincott Williams & Wilkins, 2006

In any attempt to predict growth, it is necessary to initially understand the normal growth of the lower limbs. In the lower extremities, the femur and the tibia contribute 54% and 46% of the total length at skeletal maturity. Four growth plates (proximal and distal femur and tibia, respectively) and the foot are responsible for growth, but the main gain in length takes place around the knee joint ([13]; Fig. 1). The total growth rate and the growth of the extremities decrease from birth, precisely until adolescence, when the pubertal growth spurt begins. The lower limbs gain an average of 3.2 cm per year after age 5 until puberty (2 cm per year from the femur and 1.5 cm per year from the tibia). By the beginning of puberty (Tanner stage 2 and skeletal age of 13 years in boys and 11 years in girls), the remaining growth of the lower extremities averages 10 cm in boys and 9 cm in girls before skeletal maturity is reached. Lower-limb growth rate accelerates from 3.2 to 5 cm per year at the peak of puberty [14, 15]. Menelaus defined the end of growth by the age of 14 years for girls, and 16 years for boys, respectively. Dimeglio found in both sexes that growth stops 6 months earlier in Risser 1 [16]. These formulas only apply if puberty occurs in the normal age range.

To estimate the anticipated final height of an infant, a method of calculating the mean parental height can be used. The height of the father + the height of the mother in cm is divided by 2, then 6.5 cm is added for boys, and 6.5 cm is subtracted for girls, respectively. Most children have a projective adulthood height that is within 10 cm or two standard deviations of their mean parental height. A projected adult height that deviates from the mean parental height by more than 10 cm points to a possible abnormal condition [17]. Nevertheless, neither the expected height, nor projected height is a height prognosis.

**Fig. 2:**
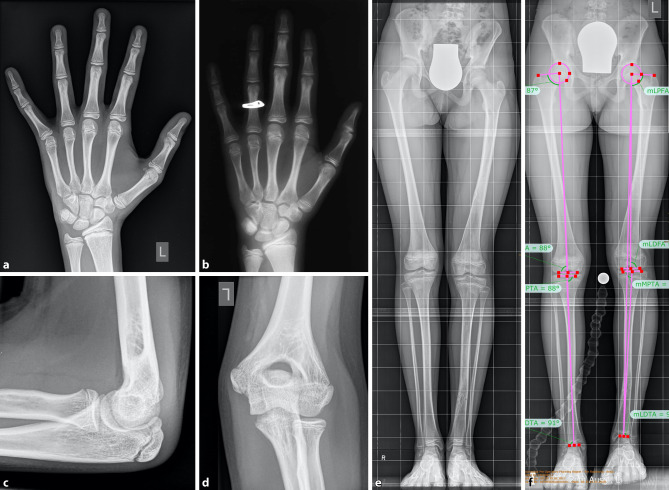
Example of bone age determination. A girl with chronological age of 12 years and 10 months. **a** Left hand X‑ray and **b** reference X‑ray according to the Greulich and Pyle atlas for a bone age of 11 years in girls. **c** Lateral and **d** antero/posterior (a/p) left elbow X‑ray to determine the bone age according to the Sauvegrain method. The evaluation results in 24.5 points corresponding to a bone age of 11.5 years in girls. The difference between the girls chronological age and the bone age is > 1 year speaking for a retarded bone age. **e** Full standing a/p bilateral lower extremity radiograph with a leg length discrepancy of −2 cm on the left side. **f** Preoperative X‑ray with 2 cm block under the left foot to equalize leg length discrepancy, and limb alignment measurements

Chronological age is based on the actual years of age. Skeletal (bone) age is a maturity indicator which is based on a set of radiographic “norms” that allow us to make predictions about the future growth. The Greulich and Pyle atlas describes stages of ossification on a plain dorso/palmar (d/p) left hand x‑ray including the wrist ([18]; Fig. 2). Based on the Greulich and Pyle atlas, Bailey and Pinneau developed tables for prediction of adult height based on the bone age. Data of the Berkley Growth Study were used including 103 girls and 89 boys measured and x‑rayed from 8 years to 18 years of age once every six months (with occasional exceptions) [19].

The Tanner–Whitehouse and the short hand bone age method are other techniques for assessing the bone age in a left hand radiograph [20, 21]. The distinct features of ossification in the pediatric skeleton can be used to assign a bone age, and correlate the findings with the chronological age of the child. If the bone age appears younger than the actual age, a retarded bone growth can be detected. Vice versa, if the skeletal findings are assigned to an older bone age, accelerated bone growth is present. During the two years of pubertal growth spurt, the Sauvegrain method has been proven to be a reliable method. The Sauvegrain technique determines bone age from a/p and lateral left elbow radiographs using a 27-point scoring system (Fig. 2). The elbow is defined by distinct developmental series of its ossification centers, beginning at age 9 in girls and age 11 in boys. Fusion of the growth centers of the elbow is finished at the age of 13 in girls and 15 in boys [22]. The findings and differences between bone age and chronological age have to be taken into account for planning of surgical limb length corrections (i.e., epiphysiodesis). 50% of children have an accelerated or retarded skeletal age [14]. A bone age that deviates more than two standard deviations from the mean age is probably attributable to a pathologic state [17].

Based on the data from Anderson, Green and Messner, several methods for growth prediction have been described and used [23]. These data include femoral and tibial lengths of children (boys and girls) from 1 year to skeletal maturity according to chronological age. For example, in order to make a simple calculation to determine the expected leg length, or leg length difference, the current length of the femur and the tibia of the normal leg is compared with the measurements by Anderson (Green–Anderson growth remaining charts). According to age and gender, the appropriate percentile on the chart can be defined and the predicted length at the time of skeletal maturity can be read from the percentiles. Moseley converted these data into a table with straight lines (Moseley Straight-Line Graph), so that the predicted leg length can be read from the graph by plotting the current leg lengths and skeletal age [24]. Menelaus described a simplified method for growth estimation, based on their own calculations, stating that the lower limb grows 23 mm per year, most of that from the knee (15 mm) ([25]; Fig. 1). The currently most popular method is the Multiplier method. Based on data from Anderson and Green, as well as a data set from Maresh [26], which contains radiologically measured lengths of femora and tibiae of infants between 0 and 1 year of age, a multiplier (M = “multiplier”) has been calculated, which results from the expected length of the bone at skeletal maturity (Lm) divided by the length at the time of the current age (L): M = Lm/L. Residual growth or expected LLD can be calculated by use of a simple formula [27].

The accuracy of the calculation is poor across all methods with a significant rate of false predictions between 10 and 27% with over 2 cm deviation of the definitive growth from the predicted value [28]. The treating pediatric orthopedic surgeon should also keep in mind that simple calculation errors have been proven to occur in 18% of instances [29]. In a direct comparison of prediction accuracy, the original Green–Anderson growth-remaining method showed the greatest correlation between expected and final LLD after epiphysiodesis. Nevertheless, all methods generated an overcorrected value [30].

## Timing of epiphysiodesis

Considering the Anderson–Green graph, the average residual growth of the knee at the onset of puberty is about 5 cm (3 cm femoral and 2 cm tibial). Based on the simplified method by Menelaus, 3 years of growth are expected from the onset of puberty. The beginning of puberty is defined by the bone age of 13 years for boys and 11 years for girls. With 1.5 cm growth resulting from the distal femoral (9 mm) and proximal tibial (6 mm) physis, we expect a 4.5 cm growth remaining at the knee. The Dimeglio method calculates growth at the level of the knee as 2 cm per year, 11 mm from the distal femur and 9 mm from the proximal tibia (Fig. 1). In contrast to Menelaus, Dimeglio calculated the time for growth remaining as 2.5 years after the onset of puberty. Nevertheless, the final results are approximately the same (Dimeglio 2 cm × 2.5 years = 5 cm) [14].

With regard to the timing of epiphysiodesis, it is necessary to emphasize that 2.6 cm of the remaining growth happens in the first year after the onset of puberty during the growth spurt. If growth arrest is planned for the correction of an expected extreme tall stature, epiphysiodesis should be performed even before the start of puberty, in order to achieve larger amounts of height reduction. Correction of limb length discrepancy must be planned in accordance with the origin of the deformity (femur/tibia/both), and following a meticulous calculation of the predicted growth remaining. The aim is to create two legs of the same length or a residual LLD < 1 cm, and to bring the flexion–extension axis of the knee joint to the same height. Epiphysiodesis is the method of choice for correcting a leg length difference of up to 5 cm ([14, 31]; Table 2). Some simple principles can help to reduce preoperative error for LLD correction: (1) rigorous and meticulous repeat measurements; (2) rigorous analysis of the data obtained; (3) perfect evaluation of bone age using left elbow plus hand radiographs (accelerated or retarded bone age must be taken into account); and (4) the decision for epiphysiodesis should always be taken at the beginning of puberty [14].

**Table 2:** Planning and timing of epiphysiodesis to correct leg length discrepancy (LLD) < 5 cm at the beginning of puberty after determining the skeletal age [14]

LLD	Epiphysiodesis location	Timing
*5* *cm*	Femur + tibia	Beginning of puberty (skeletal age of 13 for boys and 11 for girls)
*4* *cm*	Femur + tibia	Beginning of puberty + 6 months
*3* *cm*	Femur	Beginning of puberty
*2* *cm*	Femur	Beginning of puberty + 1 year
*2* *cm*	Tibia	Beginning of puberty

## Principles and techniques of epiphysiodesis

**Fig. 3:**
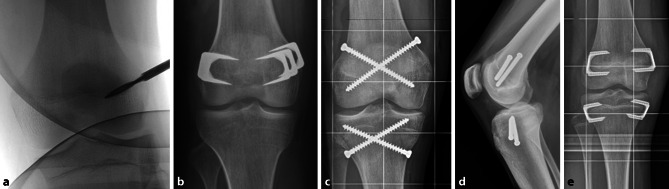
Currently used epiphysiodesis techniques. **a** Percutaneous Canale epiphysiodesis, **b** Blount staples, **c,** **d** percutaneous epiphysiodesis using transphyseal screws (PETS), **e** new implant RigidTack^TM^

The principle of epiphysiodesis (ED) is to achieve a temporary or permanent growth arrest at the physis. In 1933, Dr. Dallas Phemister introduced the first surgical method to arrest the growth of the physis. According to this uninstrumented technique, a rectangular piece of bone including a part of the growth plate is osteotomized, rotated 180° and reinserted. This technique forms an osseous connection in the region of the growth plate and is therefore a definitive procedure. Performed on both sides of the growth plate (medial and lateral), this leads to a permanent growth arrest by creation of a bony bridge [32]. Due to the invasiveness of this surgical technique, modifications in terms of a percutaneous epiphysiodesis gained popularity in the 1980s. Bowen and Johnson described a technique in which the peripheral third of the physis is removed with a curette using minor incisions [33]. The originally described procedure by Canale uses a drill and pneumatic burrs to ream the growth plate. Through minimal incisions, a drill is passed several times through the physis in order to destroy the physis (Fig. 3a). Afterwards a curette is used to remove the growth plate. Approximately 50% of the growth plate should be removed to ensure growth arrest [34].

Haas discovered in a series of animal experiments that a reversible slowing down of physeal growth is possible by implanting a wire loop in the area of the distal femoral physis. He also noticed that growth resumes as soon as the wire loop breaks. Further development using this principle resulted in the use of staples for temporary growth arrest. Blount popularized this technique and published first clinical results. Three extraperiosteal staples are placed across the lateral and medial physis to create a temporary arrest (Fig. 3b). Growth would resume after removal of the staples [35]. Nevertheless, the promising idea of a temporary growth arrest was abandoned after several reports of permanent growth arrest, angular deformity, or rebound growth after staple removal. Therefore this technique is considered a permanent epiphysiodesis [13] (Table 3). Other methods include the placement of transphyseal screws (percutaneous epiphysiodesis using transphyseal screws—PETS (Fig. 3c, d), described by Metaizeau et al. [36]. This method using fully threaded stainless steel screws may be more effective in the distal femur compared with the proximal tibia [37].

**Table 3:** Overview of epiphysiodesis (ED) techniques [38]

Technique	Temporary (t), permanent (p)	Instrumented	Invasiveness	Associated problems/complications
Phemister	p	n	+++	Permanent, precise planning mandatory, invasiveness, older children
Blount	t < p	y	++	Implant associated complications, mechanical axis deviations, metal removal
Percutaneous ED	p	n	+	Permanent, precise planning mandatory
PETS	t > p	y	+	Temporary?, metal removal, valgus deformity after tibial PETS
Tension band plating	t	y	++	Not recommended due to poor correction
New implants (i.e., rigid tack)	t > p?	y	++	Evidence missing

*PETS* percutaneous epiphysiodesis using transphyseal screws, *y* yes, *n* no

## Evidence in LLD

In general, epiphysiodesis is a reliable and safe procedure for LLD treatment. Makarov et al. reported an overall complication rate among 863 treated children of 7%. The development of angular deformity is a potential complication, especially in congenital LLD, younger patients, and larger limb length inequalities [39]. Campens et al. compared three ED techniques (Phemister, Canale and a transphyseal screw technique) in a retrospective series over a 21 year period including 80 patients. They found no statistically significant difference between the techniques concerning their efficiency in correction of LLD. Percutaneous epiphysiodesis using a transphyseal screw (PETS) appeared to be the best technique regarding a shorter mean operative time and hospital stay, as also postoperative pain and recovery of ambulation. Complication rates were comparable among the three techniques [40]. Burger et al. reported on results using the Canale epiphysiodesis for idiopathic LLD. They found a mean correction of 13.3 mm (LLD 21.2 mm preoperative and 7.9 mm postoperative) [41]. Ilharreborde et al. reported on 45 children with a mean bone age of 12.7 years (range 8.5–15 years) who were treated by PETS. Final loss of growth at maturity reached only 66% of the predicted preoperative correction. The PETS technique was safe at the femur, but at the tibia a significant rate of complications were observed, including the development of valgus deformity in 20% of patients; 18% of patients (eight) underwent reoperation and seven of these revisions involved the tibia. They concluded that PETS should be abandoned at the tibia. The overall screw removal rate was 60%, but failed in 18.5% due to screw breakage during removal. In these patients fully threaded cannulated stainless steel screws were used [37]. Siedhoff et al. achieved a mean LLD correction of 1.6 cm with temporary epiphyseodesis using mainly Blount staples [42]. Mechanical axis deviation following epiphysiodesis has been associated with the use of Blount staples. In a case series published by Gorman et al., 50% of patients exhibited a shift in the mechanical axis of > 1 cm, and 33% of patients had a clinically relevant mechanical axis zone change. Most of the zone changes occurred in patients with proximal tibial staple ED resulting in a varus deformity [43]. New implants for guided growth (i.e., tension band plating with the 8‑plate) were initially intended for angular deformity correction; nevertheless attempts were made to correct LLD. Based on the current literature, the application of tension band plates for LLD reduction cannot be recommended due to the lower correction potential with higher complication and revision rates [44–47]. Other implants designed for temporary ED (i.e., RigidTack^TM^) (Fig. 3e) are on the market and have already been tested in animal models, but clinical data on efficacy are still missing [48]. Residual LLD after growth arrest may be treated alternatively by lengthening procedures.

## Evidence in tall stature

Whereas therapy with high doses of sex steroids have been used in the past for manipulation of excessive final height , during recent years, ED has increasingly been recommended in case of subjective psychosocial stress [49]. Nevertheless, since tall stature is an “orphan disease”, mainly small case series of treated patients have been analyzed.

In 15 very tall boys (boys with a predicted final height of > 205 cm), the use of bilateral percutaneous pan genu epiphysiodesis (femur + tibia + fibula) has been reported to decrease final height in adulthood by an average of 7 cm calculating the expected final height minus the definitive postoperative final height. Indeed, this study included a control group of patients without surgery and found that final height was overestimated by 2.3 cm, which is in accordance to other similar studies. Accounting for this overestimation, the definitive height reduction was about 5 cm in the study by Odink et al. ED was undertaken at a mean bone age of 13.9 years. Chronological age at surgery was 15.2 years (range 13.7–17.6 years) [50]. Benyi et al. reported on 12 girls and 9 boys with bilateral ED. Growth reduction resulted in minus 4.1 ± 0.7 cm in treated girls and minus 6.4 ± 0.7 cm in treated boys corresponding to a 33.6 ± 3.4% and 33.6 ± 4.2% reduction of remaining growth, respectively. No major complications in terms of varus or valgus deformity or severe LLD occurred [49]. In general, there is no recommendation for the perfect age to correct expected extreme tall stature. The timing should be based on growth predictions, puberty and the desired height reduction.

**Fig. 4:**
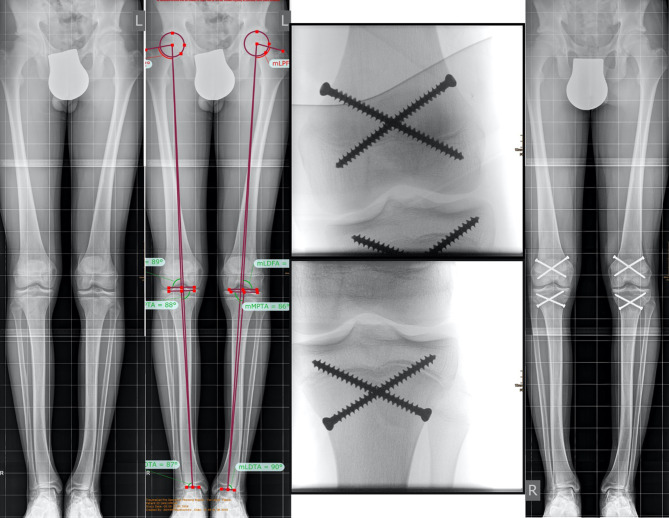
Example of bilateral percutaneous epiphysiodesis using transphyseal screws (PETS) to reduce excessive predicted height. **a** Full standing anterior/posterior bilateral lower extremity radiograph of a boy with familial tall stature. **b** Preoperative limb alignment analysis. Growth prediction resulted in a predicted final height of 208 cm. **c,** **d** Intraoperative a/p x-rays of the left distal femur and tibia; **e** follow-up X‑ray after completed growth with a final height of 197.5 cm

A Dutch multicenter study evaluated the final adult height, complications, knee function and patient satisfaction in patients treated by bilateral ED with constitutional tall stature. In all, 77 adolescents and 60 controls were included. The final height was reduced by 7.0 cm (±6.3 cm) compared with the predicted height in boys, and 5.9 cm (±3.7 cm) in girls, respectively. Short-term complications included wound infections in 4% of patients, and long term complications were seen in 2.6% including one valgus deformity and one prominent fibular head. Good knee function scores and a very high satisfaction rate of 97% could be observed [51] (Fig. 4).

## Conclusion

Epiphysiodesis for the treatment of tall stature and limb length discrepancy is a safe and efficient method. The decision for ED in tall stature should be made after careful multidisciplinary consultation and on the basis of subjective psychosocial deprivation. Especially percutaneous ED techniques (instrumented and uninstrumented) have low complication rates and high satisfaction rates. Growth prediction is susceptible for error and overestimation especially in excessively tall children. Therefore, preoperative evaluation of bone age and appropriate timing of ED are the key factors for success.
